# The change of triglyceride-glucose index may predict incidence of stroke in the general population over 45 years old

**DOI:** 10.1186/s12933-023-01870-z

**Published:** 2023-06-09

**Authors:** Yaoling Wu, Yongbiao Yang, Jinsheng Zhang, Shuo Liu, Weiduan Zhuang

**Affiliations:** 1 Shantou University Medical College, Shantou, Guangdong China; 2 Neurology Department, First Affiliated Hospital of Shantou University Medical College, No.57, ChangPing Road, JinPing District, ShanTou City, 515041 Guangdong Province China

**Keywords:** Triglyceride-glucose index, Stroke, Association, CHARLS, K-means clustering

## Abstract

**Background:**

Stroke has been found to be highly correlated with the triglyceride-glucose (TyG) index. The relation between the TyG index changes and stroke, however, has seldom been reported, and current researches mentioning the TyG index concentrate on individual values. We aimed to investigate whether the level and the change of TyG index was associated with the incidence of stroke.

**Methods:**

Sociodemographic, medical background, anthropometric and laboratory information were retrospectively collected. Classification was conducted using k-means clustering analysis. Logistic regressions were to determine the relationship between different classes with changes in the TyG index and incidence of stroke, taking the class with the smallest change as a reference. Meanwhile, restricted cubic spline regression was applied to examine the links of cumulative TyG index and stroke.

**Results:**

369 (7.8%) of 4710 participants had a stroke during 3 years. Compared to class 1 with the best control of the TyG Index, the OR for class 2 with good control was 1.427 (95% CI, 1.051–1.938), the OR for class 3 with moderate control was 1.714 (95% CI, 1.245–2.359), the OR for class 4 with worse control was 1.814 (95% CI, 1.257–2.617), and the OR for class 5 with consistently high levels was 2.161 (95% CI, 1.446–3.228). However, after adjusting for multiple factors, only class 3 still had an association with stroke (OR 1.430, 95%CI, 1.022-2.000). The relation between the cumulative TyG index and stroke was linear in restricted cubic spline regression. In subgroup analysis, similar results were shown in participants without diabetes or dyslipidemia. There is neither additive nor multiplicative interaction between TyG index class and covariates.

**Conclusion:**

A constant higher level with worst control in TyG index indicated a higher risk of stroke.

## Introduction

Insulin resistance (IR), a new risk factor for stroke, is considered to be an early manifestation of type 2 diabetes, which is present not only in diabetes but also in many non-diabetic patients [1]. It is closely related to risk factors for cerebrovascular disease, such as atherosclerosis, hypertension, atrial fibrillation, coronary heart disease and type 2 diabetes [2–5].

There are a number of methods to assess IR and the gold standard is the hyperinsulinemic glucose clamp (HEC) [6]. But HEC requires intravenous infusion of glucose and insulin and multiple blood samples [7]. The procedure is complex and costly and is not widely used in clinical practice. HOMA-IR (Homeostasis model assessment of IR) is widely used and has been shown to be effective in predicting the occurrence of cardiovascular and cerebrovascular diseases [7–12]. However, it is necessary to measure fasting insulin levels in patients, which is of limited clinical use. The triglyceride-glucose (TyG) index, as a simple alternative surrogate of IR, is easily obtained from clinical laboratory test results and is associated with the occurrence and recurrence of stroke. Further research has shown that the TyG index is superior to HOMA-IR in predicting stroke [13, 14].

Nevertheless, there are fewer studies dealing with change of TyG index and stroke incidence. This article aimed to evaluate whether the level and the change in TyG index predicted the incidence of stroke using data from the “China Health and Retirement Longitudinal Study (CHARLS)”.

## Methods

### Study population

**Fig. 1:**
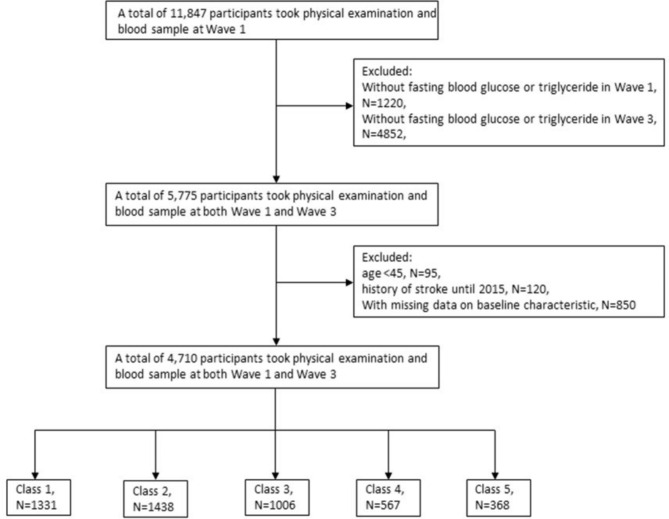
Flowchart of the study population

CHARLS is a nationally representative longitudinal survey of the middle-aged and older population (≥ 45 years) conducted by the National School of Development at Peking University. Detailed information on the study population has been reported in other publications [15]. To date, the nationwide baseline study has been conducted in 2011–2012, with Wave 2 in 2013, Wave 3 in 2015 and Wave 4 in 2018. Blood samples were also collected at baseline (including 11,847 participants) and wave 3 (including 13,420 participants). For this analysis, participants had to be aged 45 years and older. Complete data on fast blood glucose (FBG) and triglycerides (TG) were required. People were excluded if they had had a stroke before 2015. 4,710 respondents had complete data in both 2012 and 2015 in this cohort (68.7%) and were considered eligible to participate finally (Fig. 1).

### Data assessment

#### The change of TyG index and stroke

Stroke was the main outcome of this study. Individuals who self-reported “yes” to the question of “Have you been diagnosed with stroke by a doctor?” or had specific answers with questions of “Treat stroke by Take Chinese Traditional Medicine/Take Western Modern Medicine/Physical Therapy/Acupuncture and Moxibustion/Occupational Therapy/None of the Above” were regarded as people with stroke.

The exposure of this study was the change of TyG index respectively collected in 2012 and 2015. The TyG index was calculated by the formula ln[TG (mg/dl) × FBG (mg/dl)/2] [16]. The cumulative TyG index was determined by the expression: (TyG_2012_ + TyG_2015_)/2*time_(2015−2012)_ [17].

### Data collection

Baseline demographic information (age, sex, Hukou, education and marital status), basic anthropometric measurements (systolic blood pressure (SBP), diastolic blood pressure (DBP) and body mass index (BMI)) and potential risk factors (hypertension, diabetes, dyslipidemia, heart problems, smoking status and alcohol drinking status) were reviewed.  Primary prevention included antihypertensive treatments, lipid-lowering treatments and hypoglycaemic treatments. Laboratory examination contained fast blood glucose (FBG), total cholesterol (TC), triglyceride (TG), high-density lipoprotein cholesterol (HDL), low-density lipoprotein cholesterol (LDL) and glycosylated Hemoglobin, Type A1c (HbA1c) [18].

### Statistical analysis

**Fig. 2:**
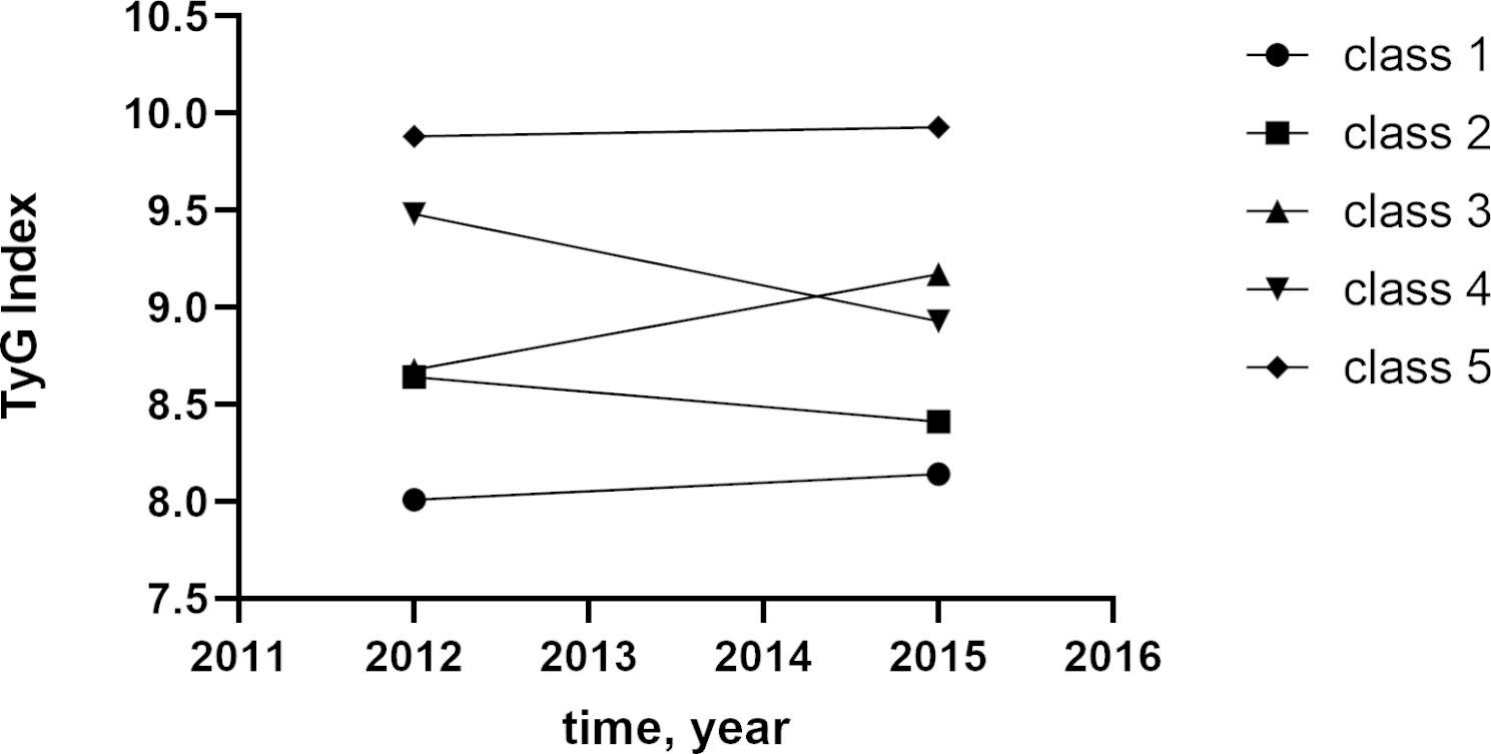
The TyG index clustering by k-means clustering

The data set of the 3-year transition of the TyG index was analyzed and classified into 5 classes using K-means clustering. K-means clustering is a technique that has the goal of dividing N observations into K clusters. Each observation is assigned to the cluster with the closest mean value, which serves as the prototype of the cluster. As the number of categorized classes was increased, the maximum number of classes recruited was 5, with each class containing no less than two data. When the number of clusters equaled to 5, the effect of k-means clustering was better than others. Further categorization into final classes was performed for each of the 5 classes that met the criteria below: Class 1, the TyG index was from 8.01 in 2012 to 8.14 in 2015, representing better control of TyG index; Class 2, the TyG index was from 8.64 in 2012 to 8.41 in 2015, representing good control of TyG index; Class 3, the TyG index was from 8.68 in 2012 to 9. 17 in 2015, representing moderate control of TyG index; class 4, the TyG index was from 9.48 in 2012to 8.93 in 2015, from high level to low level, representing worse control for its higher cumulative TyG index than class 3; class 5, the TyG index was from 9.88 in 2012 to 9.93 in 2015, representing worst and terrible control of TyG index (Fig. 2).

Descriptive statistics (means and standard deviations, SD, for continuous data and percentages for categorical data) were used to report basic characteristics. The t-test or Mann-Whitney U test for continuous variables and the chi-squared test or Fisher’s exact test for categorical variables were used to analyze differences in baseline characteristics between classes. Univariate and multivariate logistic regression analysis were performed with factors important for stroke incidence. A restricted cubic spline model was then carried out for examining the shape of the correlation between cumulative TyG index and stroke. We chose four knots at 20th, 40th, 60th and 80th. Also, we performed several subgroup analyses and interaction analysis to discover potential impact factors. The relative excess risk due to interaction (RERI), the attributable proportion due to interaction (AP) and the synergy index (SI) were utilized to evaluate the additive interaction. The effects of interaction analysis between TyG index classes and covariate were reported by ORs (95%CI). Statistical analyses were performed using R version 4.0.3 software (http://www.R-project.org/). Two-sided P < 0.05 was considered statistically significant.

## Results

### Baseline characteristics of study participants

This study included 4,710 participants with an average age of 58.73 ± 8.88 years and 44.48% males. The mean TyG index was 8.67 ± 0.64 in 2012 and 8.68 ± 0.62 in 2015 and the mean cumulative TyG index was 26.02 ± 1.69. Compared with class 1, participants in the other classes had fewer current smokers and drinkers, higher BMI, SBP and DBP, a higher prevalence of hypertension, diabetes, dyslipidemia and heart disease, and had higher FBG, TC, TG, LDL, HbA1 levels and lower HDL levels (Table 1).

**Table 1:** Baseline characteristics according to the change of TyG index

	Total	Class1	Class 2	Class 3	Class 4	Class 5	P value
n	4,710	1,331	1,438	1,006	567	368	
Sex(male)	2,095 (44.48)	718 (53.94)	641 (44.58)	368 (36.58)	234 (41.27)	134 (36.41)	< 0.001
Age	58.73 (8.88)	58.82 (9.35)	59.07 (8.80)	58.17 (8.67)	59.01 (8.41)	58.12 (8.60)	0.073
Education level							0.812
Primary school or lower	3,336 (70.83)	956 (71.83)	1,017 (70.72)	699 (69.48)	404 (71.25)	260 (70.65)	
Secondary school or higher	1,374 (29.17)	375 (28.17)	421 (29.28)	307 (30.52)	163 (28.75)	108 (29.35)	
Current married	4,187 (88.90)	1,176 (88.35)	1,272 (88.46)	892 (88.67)	506 (89.24)	341 (92.66)	0.193
Hukou							< 0.001
Agriculture	3,997 (84.86)	1,169 (87.83)	1,231 (85.61)	844 (83.90)	462 (81.48)	291 (79.08)	
Others	713 (15.14)	162 (12.17)	207 (14.39)	162 (16.10)	105 (18.52)	77 (20.92)	
Smoking status							< 0.001
Never	2,944 (62.51)	760 (57.10)	893 (62.10)	668 (66.40)	376 (66.31)	247 (67.12)	
Former	392 (8.32)	106 (7.96)	118 (8.21)	84 (8.35)	48 (8.47)	36 (9.78)	
Current	1,374 (29.17)	465 (34.94)	427 (29.69)	254 (25.25)	143 (25.22)	85 (23.10)	
Drinking status							< 0.001
Never	2,929 (62.19)	756 (56.80)	906 (63.00)	660 (65.61)	358 (63.14)	249 (67.66)	
Former	289 (6.14)	83 (6.24)	87 (6.05)	61 (6.06)	40 (7.05)	18 (4.89)	
Current	1,492 (31.68)	492 (36.96)	445 (30.95)	285 (28.33)	169 (29.81)	101 (27.45)	
SBP	129.64 ± 0.93	124.66 ± 9.90	129.71 ± 1.26	131.40 ± 20.80	133.82 ± 20.39	136.18 ± 0.58	< 0.001
DBP	75.51 ± 11.90	72.87 ± 11.56	75.09 ± 11.87	76.79 ± 11.92	77.84 ± 11.40	79.67 ± 11.70	< 0.001
BMI	23.67 ± 3.82	22.10 ± 3.36	23.35 ± 3.91	24.54 ± 3.49	25.07 ± 3.68	26.04 ± 3.53	< 0.001
Hypertension	1,217 (25.84)	222 (16.68)	343 (23.85)	296 (29.42)	202 (35.63)	154 (41.85)	< 0.001
Dyslipidemia	483 (10.25)	72 (5.41)	120 (8.34)	123 (12.23)	84 (14.81)	84 (22.83)	< 0.001
Diabetes	267 (5.67)	32 (2.40)	51 (3.55)	56 (5.57)	51 (8.99)	77 (20.92)	< 0.001
Antihypertensive treatments	908 (19.28)	151 (11.34)	240 (16.69)	233 (23.16)	128 (22.58)	156 (42.39)	< 0.001
Lipid-lowering treatments	295 (6.26)	43 (3.23)	61 (4.24)	76 (7.55)	63 (11.11)	52 (14.13)	< 0.001
Hypoglycemic treatments	163 (3.46)	16 (1.20)	26 (1.81)	33 (3.28)	58 (10.23)	30 (8.15)	< 0.001
Heart Disease	554 (11.76)	119 (8.94)	164 (11.40)	120 (11.93)	81 (14.29)	70 (19.02)	< 0.001
FBG	108.90 ± 32.70	97.14 ± 12.80	103.90 ± 16.69	122.65 ± 45.55	104.6 ± 18.66	152.17 ± 63.85	< 0.001
TC	194.00 ± 38.21	180.00 ± 33.05	192.70 ± 36.09	198.40 ± 37.28	209.40 ± 40.95	213.90 ± 41.89	< 0.001
TG	129.60 ± 93.59	64.42 ± 15.23	113.40 ± 31.79	118.78 ± 34.64	226.20 ± 74.64	309.55 ± 179.05	< 0.001
HDL	51.16 ± 15.16	59.59 ± 14.90	51.97 ± 13.99	49.55 ± 13.03	42.09 ± 11.03	35.90 ± 9.91	< 0.001
LDL	117.30 ± 35.05	109.40 ± 28.70	119.70 ± 32.46	126.37 ± 34.59	119.80 ± 41.18	107.66 ± 46.74	< 0.001
HbA1c	5.29 ± 0.81	5.09 ± 0.43	5.17 ± 0.52	5.23 ± 0.62	5.55 ± 1.10	6.17 ± 1.66	< 0.001
TyG_2012_	8.67 ± 0.64	8.01 ± 0.25	8.64 ± 0.26	8.68 ± 0.30	9.48 ± 0.33	9.88 ± 0.57	< 0.001
TyG_2015_	8.68 ± 0.62	8.14 ± 0.35	8.41 ± 0.26	9.17 ± 0.30	8.93 ± 0.40	9.93 ± 0.42	< 0.001
Cumulative TyG	26.02 ± 1.69	24.23 ± 0.60	25.58 ± 0.48	26.77 ± 0.68	27.61 ± 0.64	29.72 ± 1.04	< 0.001

### Odds ratios for incident stroke

**Fig. 3:**
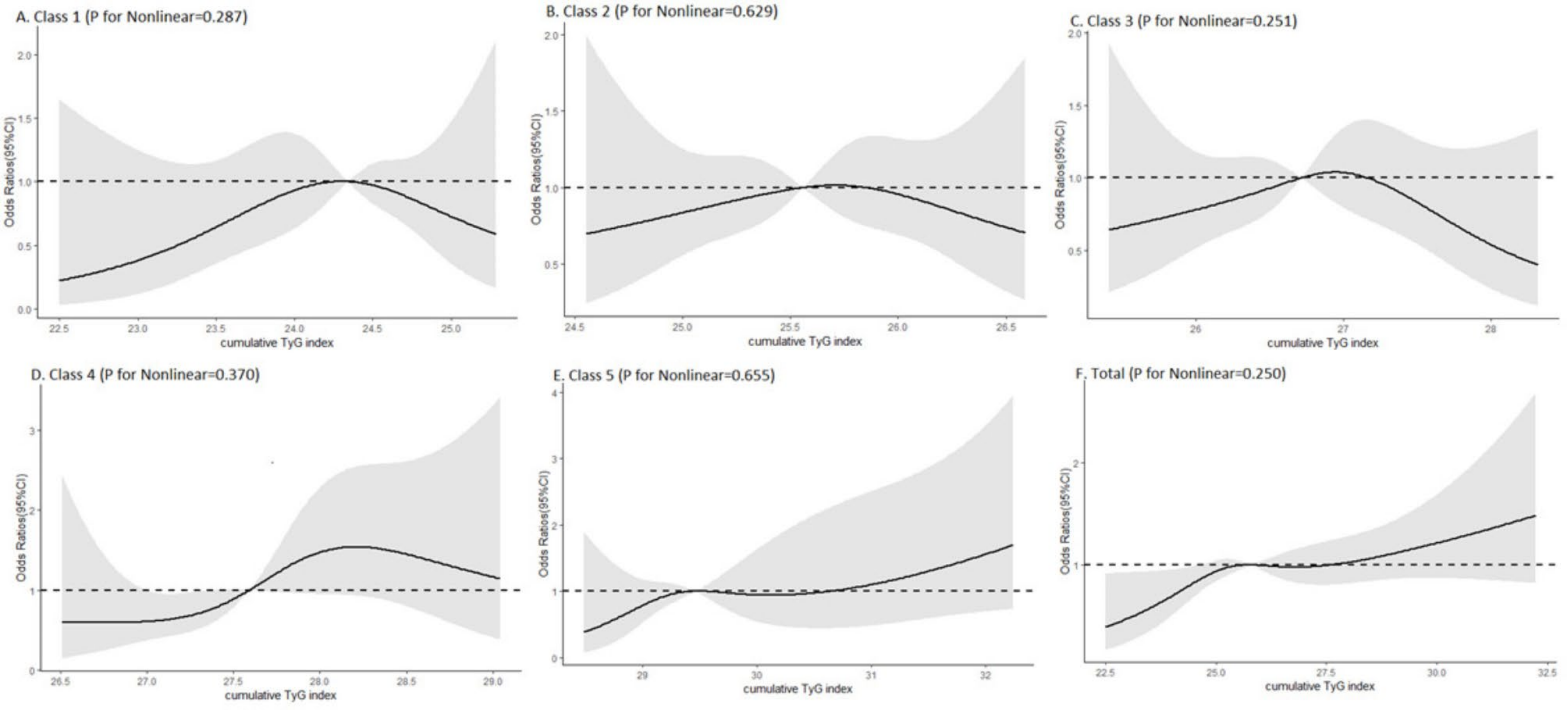
Cubic model of the association between different classes and cumulative TyG index after adjusting for sex, age, education, marital status, Hukou, smoking status, drinking status, SBP, DBP, BMI, history of hypertension, dyslipidemia, diabetes, heart disease, antihypertensive treatments, lipid-lowering treatments and hypoglycemic treatments

After 3 years, 369 (7.8%) participants had developed a stroke (Table 2). Comparing to class 1, the ORs for incident stroke were 1.427 (1.051, 1.938) for class 2, 1.714 (1.245, 2.359) for class 3, and 1.814 (1.257, 2.617) for class 4. It was interesting that class 3 and class 4 had approximate TyG index but different tendency, and class 4 had a higher risk of stroke than class 3. Class 5, with the worst control of TyG index, had the highest risk to develop new-onset stroke (OR:2.161, 95% CI, 1.446, 3.228), even though both class 1 and class 5 had elevating TyG index. With adjustment for sex, age, education, marital status, Hukou, smoking status, drinking status, SBP, DBP, BMI, history of hypertension, diabetes, dyslipidemia, heart disease, antihypertensive treatments, lipid-lowering treatments and hypoglycemic treatments (model 3), only class 3 had an increased risk (OR:1.423, 95% CI, 1.017, 1.992). In the restricted cubic spline regression models, the correlation between cumulative TyG index and risk of incident stroke was linear shown in Fig. 3  (P = 0.250). The risk of stroke was increasing with each increase in the cumulative TyG index above 27.64 (OR 1.002, 95% CI, 0.807, 1.246). And with the raising of the cumulative TyG index over 28.20 (OR 1.538, 95% CI, 0.932, 2.538) in class 4 and over 30.66 (OR 1.003, 95% CI, 0.442, 2.276) in class 5, participants were more likely to have an incidence of stroke.

**Table 2:** Logistic regression analysis for the association between different classes and stroke

		Crude	Model 1	Model 2	Model 3
	Case	OR(95%CI)	OR(95%CI)	OR(95%CI)	OR(95%CI)
Total	369 (7.8%)	-	-	-	-
Class1	73 (5.5%)	ref	ref	ref	ref
Class2	110 (7.6%)	1.427 (1.051, 1.938)	1.440 (1.059, 1.958)	1.301 (0.952, 1.777)	1.285 (0.939, 1.758)
Class3	91 (9.0%)	1.714 (1.245, 2.359)	1.787 (1.294, 2.469)	1.498 (1.074, 2.088)	1.430 (1.022, 2.000)
Class4	54 (9.5%)	1.814 (1.257, 2.617)	1.846 (1.277, 2.669)	1.487 (1.015, 2.177)	1.366 (0.927, 2.012)
Class5	41 (11.1%)	2.161 (1.446, 3.228)	2.263 (1.509, 3.393)	1.710 (1.119, 2.612)	1.391 (0.895, 2.161)

Mode 1, adjusted for sex and age; Model 2, adjusted for sex, age, education, marital status, Hukou, smoking status, drinking status, SBP, DBP and BMI; Model 3, adjusted for factors in model 2 and history of hypertension, dyslipidemia, diabetes, heart disease, antihypertensive treatments, lipid-lowering treatments and hypoglycaemic treatments.

### Subgroup analyses

We performed subgroup analysis to stratify the relevance between the change in the TyG index and stroke as shown in Table 3. Younger male participants with agriculture Hukou were at-risk population. A positive association was found in patients without diabetes, dyslipidemia or heart disease. No interaction was found between TyG index classes and subgroup variables (Table 4). The SI of education level or marital status in addictive interaction analysis was null because of meaningless statistical value. 

**Table 3:** Subgroup analysis of different classes and stroke

	Case/Total	Class 1	Class 2	Class 3	Class 4	Class 5
Sex						
Male	171/2,095	ref	1.409 (0.911, 2.181)	2.183 (1.381, 3.450)	2.366 (1.421, 3.940)	2.195 (1.173, 4.106)
Female	198/2,615	ref	1.436 (0.932, 2.213)	1.448 (0.923, 2.272)	1.442 (0.850, 2.448)	2.129 (1.247, 3.633)
Age, Years						
≥ 60	199/2,119	ref	1.164 (0.776, 1.745)	1.609 (1.054, 2.456)	1.485 (0.906, 2.434)	1.828 (1.036, 3.225)
< 60	170/2,591	ref	1.873 (1.165, 3.012)	1.990 (1.211, 3.270)	2.371 (1.360, 4.135)	2.827 (1.575, 5.073)
Education level						
Primary school or lower	275/3,336	ref	1.248 (0.885, 1.759)	1.403 (0.973, 2.025)	1.541 (1.014, 2.342)	1.952 (1.239, 3.076)
Secondary school or higher	94/1,374	ref	2.448 (1.205, 4.972)	3.452 (1.695, 7.031)	3.354 (1.505, 7.473)	3.377 (1.394, 8.181)
Current married						
Yes	310/4,187	ref	1.688 (1.204, 2.366)	1.809 (1.264, 2.590)	2.000 (1.334, 2.998)	2.288 (1.472, 3.555)
No	59/523	ref	0.576 (0.261, 1.272)	1.423 (0.692, 2.925)	1.225 (0.499, 3.008)	2.319 (0.822, 6.547)
HuKou						
Agriculture	315/3,997	ref	1.502 (1.082, 2.084)	1.788 (1.267, 2.523)	1.988 (1.340, 2.949)	2.018 (1.280, 3.181)
Others	54/713	ref	1.019 (0.435, 2.386)	1.326 (0.564, 3.118)	1.086 (0.400, 2.947)	2.533 (1.026, 6.255)
Smoking tatus						
Never	216/2,944	ref	1.524 (1.015, 2.287)	1.593 (1.039, 2.442)	1.661 (1.042, 2.708)	2.082 (1.233, 3.517)
Former	44/392	ref	0.998 (0.389, 2.558)	1.456 (0.563, 3.766)	2.156 (0.776, 5.984)	2.602 (0.891, 7.594)
Current	109/1,374	ref	1.426 (0.830, 2.448)	2.181 (1.242, 3.828)	2.062 (1.056, 4.029)	2.084 (0.937, 4.638)
Drinking status						
Never	230/2,929	ref	1.732 (1.164, 2.576)	1.540 (1.001, 2.368)	1.682 (1.026, 2.755)	2.711 (1.658, 4.432)
Former	32/289	ref	0.719 (0.255, 2.030)	1.241 (0.450, 3.427)	1.451 (0.478, 4.403)	1.028 (0.202, 5.218)
Current	107/1,492	ref	1.112 (0.629, 1.966)	2.363 (1.370, 4.075)	2.227 (1.182, 4.194)	1.391 (0.585, 3.310)
BMI, kg/m2						
≥ 24	184/1,986	ref	1.380 (0.936, 2.033)	2.012 (1.324, 3.057)	2.327 (1.407, 3.848)	1.139 (0.477, 2.721)
< 24	185/2,724	ref	1.286 (0.770, 2.148)	1.203 (0.714, 2.027)	1.199 (0.678, 2.121)	1.961 (1.127, 3.414)
Hypertension						
Yes	165/1,217	ref	1.340 (0.787, 2.281)	1.313 (0.759, 2.270)	1.509 (0.845, 2.696)	1.839 (1.010, 3.348)
No	204/3,493	ref	1.315 (0.899, 1.922)	1.674 (1.122, 2.498)	1.491 (0.903, 2.462)	1.483 (0.804, 2.733)
Dyslipidemia						
Yes	79/483	ref	1.400 (0.600, 3.267)	1.047 (0.437, 2.508)	2.046 (0.861, 4.862)	1.522 (0.622, 3.721)
No	290/4,227	ref	1.368 (0.984, 1.904)	1.733 (1.227, 2.448)	1.459 (0.953, 2.234)	1.882 (1.170, 3.027)
Diabetes						
Yes	38/267	ref	1.157 (0.350, 3.824)	0.098 (0.011, 0.882)	1.317 (0.405, 4.279)	1.097 (0.356, 3.379)
No	331/4,443	ref	1.422 (1.035, 1.953)	1.894 (1.366, 2.627)	1.688 (1.138, 2.502)	1.927 (1.217, 3.052)
Heart disease						
Yes	72/554	ref	1.994 (0.887, 4.481)	2.157 (0.927, 5.018)	1.528 (0.579, 4.032)	2.788 (1.124, 6.912)
No	297/4,156	ref	1.315 (0.943, 1.834)	1.611 (1.138, 2.280)	1.830 (1.231, 2.722)	1.860 (1.170, 2.957)
Antihypertensive treatments						
Yes	132/908	ref	1.108 (0.605, 2.029)	1.187 (0.650, 2.029)	1.442 (0.742, 2.804)	1.263 (0.660, 2.416)
No	237/3,802	ref	1.432 (1.002, 2.048)	1.660 (1.131, 2.437)	1.793 (1.042, 3.083)	1.642 (1.035, 2.604)
Lipid-lowering treatments						
Yes	55/295	ref	2.264 (0.748, 6.849)	1.568 (0.518, 4.745)	1.608 (0.516, 5.011)	2.280 (0.734, 7.085)
No	314/4,415	ref	1.345 (0.976, 1.852)	1.643 (1.173, 2.300)	1.957 (1.249, 3.067)	1.593 (1.069, 2.374)
Hypoglycemic treatments						
Yes	25/163	ref	0.714 (0.160, 3.182)	1.094 (0.009, 0.925)	0.480 (0.124, 1.862)	0.913 (0.222, 3.751)
No	344/4,547	ref	1.451 (1.060, 1.985)	1.841 (1.330, 2.548)	2.151 (1.393, 3.323)	1.732 (1.178, 2.546)

**Table 4:** Interaction analysis of Covariates and TyG index classes

	multiplicative interaction	additive interaction		
		RERI	AP	SI
Sex	0.887 (0.759, 1.037)	-0.11 (-0.56, 0.28)	-0.07 (-0.24, 0.10)	0.83 (0.65, 1.06)
Age, Years	0.929 (0.794, 1.087)	0.02 (-0.50, 0.35)	0.01 (-0.07, 0.15)	1.02 (0.86, 1.22)
Education level	1.132 (0.947, 1.353)	0.08 (-0.30, 0.21)	0.11 (-0.07, 0.84)	NA
Current married	1.010 (0.811, 1.257)	-0.06 (-0.51, 0.12)	-0.08 (-0.42, 0.56)	NA
Hukou	1.056 (0.847, 1.316)	0.05 (-0.56, 0.47)	0.05 (-0.18, 0.53)	1.91 (0.00, 8923.50)
Smoking tatus	1.043 (0.957, 1.136)	0.04 (-0.08, 0.10)	0.04 (-0.01, 0.13)	1.38 (0.46, 4.19)
Drinking status	1.038 (0.954, 1.131)	0.02 (-0.09, 0.07)	0.02 (-0.03, 0.12)	1.88 (0.00, 1363.72)
BMI, kg/m2	0.872 (0.742, 1.025)	-0.10 (-0.69, 0.15)	-0.05 (-0.13, 0.10)	0.91 (0.78, 1.06)
Hypertension	0.990 (0.841, 1.164)	0.15 (-0.61, 0.58)	0.05 (-0.03, 0.20)	1.09 (0.90, 1.33)
Dyslipidemia	1.031 (0.840, 1.266)	0.24 (-0.94, 0.96)	0.09 (-0.01, 0.32)	1.17 (0.85, 1.61)
Diabetes	0.948 (0.714, 1.257)	0.05 (-2.46, 1.41)	0.02 (-0.13, 0.44)	1.04 (0.71, 1.53)
Heart disease	0.951 (0.776, 1.165)	0.07 (-0.96, 0.60)	0.03 (-0.09, 0.25)	1.05 (0.80, 1.39)
Antihypertensive treatments	0.941 (0.794, 1.116)	0.07 (-0.96, 0.56)	0.02 (-0.08, 0.18)	1.03 (0.86, 1.25)
Lipid-lowering treatments	0.962 (0.756, 1.225)	0.17 (-2.20, 1.42)	0.05 (-0.09, 0.32)	1.08 (0.80, 1.45)
Hypoglycemic treatments	0.853 (0.598, 1.218)	-0.19 (-6.63, 2.72)	-0.06 (-0.26, 0.49)	0.93 (0.63, 1.36)

## Discussion

We discovered from the CHARLS national data that adults over 45 had a greater risk of stroke with a constant and high TyG index. In subgroup analysis, though, patients with diabetes or dyslipedia did not exhibit the association.

Our findings were consistent with other researches that found a linear relationship between the TyG index and the incidence of stroke. In 2020, Shi revealed the independent correlation between the TyG index and ischemic stroke in the general population and indicated that this relationship was linear for the first time [19]. Subsequently, a large prospective cohort study involving patients with acute ischemic stroke showed that TyG index was associated with stroke recurrence [20]. Our restricted cubic spline model revealed the relevance between cumulative TyG and stroke, similar to Cui’s study [17]. Another study also mentioned about change of TyG index, but the change of TyG index was calculated by the TyG index minus that at baseline. They revealed that changes in TyG index independently predicted stroke [21]. However, patients with similar cumulative TyG index may lead to different prognosis like class 3 and class 4 in this study. Studies in patients with hypertension had also shown the same conclusion, and there was no gender difference [22]. An increase in the TyG index was an independent predictor of ischemic stroke in the general population [23, 24]. However, Yang showed that the TyG index was not an efficient predictor of adverse prognosis of cardiovascular and cerebrovascular diseases in non-diabetic patients receiving percutaneous coronary intervention [2]. A meta-analysis included seven cohort studies including participants from Spain, Argentina, China, South Korea and Iran assessed the association between the TyG index and incidence of cardiovascular and cerebrovascular events due to arterial atherosclerosis, and showed that TyG index might be independently related to stroke [25]. In 2022, a meta-analysis involving 11 studies yielded the same results [26].

In model 3 of the multivariate regression analysis, except for class 3, the relationship between classes and stroke risk become insignificant, but the lower limit of the confidence interval was closely to 1. There was a possibility that the results may be positive with an increase in the sample size. In the population with diabetes and dyslipidemia, there was no significant association between each class and the incidence of stroke in the subgroup analysis. The possible reason was that hypoglycemic and lipid-lowering treatments interfered with determining TyG. It was speculated that the use of hypoglycemic and lipid-lowering drugs may be beneficial in preventing the risk of stroke. In subgroup analysis, positive relationship remained in participants without diabetes and dyslipidemia.

The mechanism of insulin resistance leading to stroke has not been clarified, which mainly has the following possibilities: First, insulin resistance leads to endothelial dysfunction, the formation of foam cells and vulnerable plaques, which plays an important role in atherosclerosis. Previous studies have revealed that the TyG index is link with atherosclerosis in the general population [27, 28] and is an independent predictor of plaque progression [29–32]. At the same time, insulin resistance, as a low-grade inflammatory state, accelerates the progress of atherosclerosis and leads to the synthesis of more inflammatory markers [33, 34]. Second, insulin resistance affects platelet adhesion, activation, and aggregation [35–39], leading to stroke through arterial stenosis or occlusion. Third, insulin resistance seems to be associated with increased sympathetic nervous system activity [16] and impaired cardiac autonomic nervous function [19], which is related to the pathogenesis of acute cardiovascular and cerebrovascular diseases. Fourth, with insulin resistance are more likely to have a high waist circumference, BMI, hypertension, diabetes, cardiovascular disease and dyslipidemia history, and a high level of fasting blood glucose, triglycerides, and glycosylated hemoglobin, all of which are exact risk factors for stroke [19–21, 23, 24].

To the best of our knowledge, this is the first study to use cluster analysis to classify changes in the TyG index. Each category represented a different population, with participants with good control having the lowest risk and patients with worst control having the highest risk. In previous studies, the prediction of stroke was mostly based on one value of the TyG index, but the value was different on the other day. The second is that our research population was a representative sample of healthy people from all over the China. Our research focused on dynamic processes and added evidence to the relationship between the TyG index and stroke.

Our study had several limitations. Firstly, although the TyG index was a reliable and convenient surrogate of insulin resistance and had been shown to correlate significantly with HEC and HOMA-IR, this study did not compare the TyG index with the gold standard for insulin resistance and cannot directly explain the relationship between insulin resistance and stroke. Secondly, there may be a bias due to the exclusion of individuals without complete fasting glucose and triglyceride data. Thirdly, there were only two blood tests, and a more detailed development of the TyG index cannot be obtained. Fourthly, the study population was all from China, and the results cannot be extrapolated to other countries.

## Conclusions

In the present study, we observed that participants with higher baseline TyG index and a change of elevating TyG index may suffer an increased incidence of stroke. Hence, people with a higher TyG index and poor TyG index control, especially in those without diabetes and dyslipidemia, should pay attention to stroke prevention.

## Data Availability

Online repositories contain the datasets used in this investigation. The names of the repository/ repositories and accession number(s) can be found at: http://charls.pku.edu.cn/en.

## Abbreviations

BMI: Body mass index
CI: Confidence interval
HDL: Low-density lipoprotein cholesterol
LDL-C: Low-density lipoprotein cholesterol
OR: Odds ratio
TC: Total cholesterol
TG: Triglyceride
TyG: Triglyceride-glucose.
